# Pollution characteristics and ecological risk assessment of heavy metals in paddy fields of Fujian province, China

**DOI:** 10.1038/s41598-020-69165-x

**Published:** 2020-07-22

**Authors:** Zhiming Kang, Songliang Wang, Junhao Qin, Renyue Wu, Huashou Li

**Affiliations:** 1 College of Natural Resources and Environment/Key Laboratory of Agro-Environment in the Tropics of Agriculture Ministry of the People’s Republic of China/Guangdong Provincial Key Laboratory of Eco-Circular Agriculture, South China Agricultural University, Guangzhou, 510642 China; 2 College of Agriculture, Fujian Agriculture and Forestry University, Fuzhou, 350002 China; 3 Key Laboratory of Ministry of Education for Genetics, Breeding and Multiple Utilization of Crops, College of Agriculture, Fujian Agriculture and Forestry University, Fuzhou, 350002 China

**Keywords:** Environmental sciences, Environmental impact

## Abstract

To analyze the concentration, spatial distribution patterns, and ecological risks of heavy metals (Cd, Cr, Pb, As, Cu, Ni and Co), 272 topsoil samples (0–20 cm) were collected from paddy fields in Fujian province in July 2017. The results revealed that the mean concentration of all heavy metals exceeded the background values in Fujian province, with the mean concentration of Cd being 5.20 times higher than its background. However, these concentrations of heavy metals were lower than their corresponding national standards (GB 15618-1995). Spatially, for Cd, the high concentration areas were located mainly in southeast of Sanming city and northeast of Quanzhou city. For Pb and As, the places of highest concentration were mainly in southeast of Quanzhou city and Zhangzhou city, and the main areas of high Ni concentration were distributed southeast of Nanping city. The geo-accumulation index (${I}_{geo}$) of Cd and As were indicative of moderate contaminations, and the index of Co, Cu and Cr suggested that these were practically uncontaminated. The nemerow integrated pollution index (${P}_{n}$) showed that the entire study area was prone to a low level of pollution, but at the county level, Yongcun county and Zhaoan county are in an warning line area of pollution. According to the potential ecological risk ($RI$), the ecological risk belongs to the low risk of paddy fields in Fujian province. However, Cd should be given attention (${E}_{r}$ = 25.09), as it contributed to the majority of potential ecological risks in Fujian province.

## Introduction

The large number of industrial waste, mining byproducts, chemical fertilizers, pesticides and other synthetic chemical substances in the agroecological ecosystem has seriously threatened food security, food safety and human health around the world^1–6^. Thus, soil pollution treatment is urgently needed to solve the current problems in agricultural production. At the same time, the accumulation of heavy metals in agricultural soil is a serious problem for human health and safe food production^7–9^. Heavy metal contaminants are commonly introduced to soils through anthropogenic activities, such as mineral resources development, agrochemical use, fossil fuel consumption, poultry manure, waste disposal, and sewage irrigation^10–12^. Soil heavy metal contamination has become serious and widespread in China. According to the national communiqué of the soil pollution survey by the ministry of environmental protection of China and the ministry of land and resources in 2014^13^, some regions of soil have become heavily contaminated, and the quality of cultivated soil is particularly concerning. The total over-standard rate of soil (environmental quality standard for soils in China, GB15618-1995) in China was 16.1%. Further, the over-standard rates of Cd, Cu, Hg, As, Pb, Ni and Cr were 7.0%, 2.1%, 2.7%, 1.5%, 4.8% and 1.1%, respectively^13^. A soil pollution control plan has been developed in China to improve soil quality, ensure the quality and safety of agricultural products and protect the health of humans, such as a national action plan “Soil Ten Chapter”^14^.

However, most of the prior studies were focused on a subset of typical areas, such as chemical factories, mining and smelting areas, to measure the heavy metal concentration of the sampling sites and then to assess the pollution level and ecological risk. Previously, some researchers concluded that there is heavy metal pollution, causing concern for human health, in urban soils around an electronics manufacturing facility in Xianyang, Hubei province, China^15^. In addition, a case study conducted in Pahang, Malaysia, reported that iron ore-mining had significant effects on surface soil heavy metals pollution^16^. Meanwhile, there are some domestic studies that focus on mining regions in China, such as the gold mining area in Tongguan, Shanxi province^1^, the lead–zinc mining area in Chenzhou, Hunan province^17^, Sb mining, lead–zinc mining, and pyrite mining areas in Lengshuijiang, Hengyang, and Liuyang, Hunan province^18^, the multi-metal mining area in Shaoguan, Guangdong province^19^, and a tin-polymetallic ore field in the Guangxi Zhuang Autonomous Region^20^. However, most studies on heavy metal pollution are limited to small scale areas, such as cities, towns, villages, sub basins and field test monitoring points. For example, Lü et al. found that in mining areas of You'xi County, the concentrations of Cd, Cr, Cu, Hg, Pb, and Zn exceeded the background values of provincial and national standards^21^. And the agricultural soils from Yangshuo county, Guangxi province also were heavily contaminated with Cd, Cu, Pb and Zn, and exceeded national soil quality standards^22^. Diami et al. found that seven locations in the vicinity of active and abandoned iron ore-mining sites in Pahang, Malaysia, the concentrations of Cu was exceeded the soil guideline value at the all sampling locations^16^. Thus, it is clear that the research on heavy metal pollution of agricultural land, especially in paddy fields, is very limited on large or moderate regional scales. However, to better manage the quality of the soil environment, it is necessary to strengthen the study of heavy metal pollution in paddy soils of a large regional scale, such as a provincial level. In recent years, the evaluation of levels and risks for soil heavy metals pollution have been widely used to the methods of geo-accumulation index (${I}_{geo}$), nemerow integrated pollution index (${P}_{n}$) and potential ecological risk index ($RI$), which considers the lithology, maximum value, toxicity variance of the different heavy metals and the comprehensive effect of multiple contaminants, have been applied frequently^15,23,24^.

Rice production is an important contributor to China’s food security^25^. However, consumption of rice contaminated with toxic heavy metals has been reported in central China, such as the “Cd rice” in Hunan province^26,27^. Meanwhile, Fujian province belongs to a double-cropping rice area in China, which contributes to local food security, and approximately 6.19 × 10^5^ hectare be cultivated in 2018. Therefore, it is necessary to understand heavy metals in this context, such as their pollution levels and risks in the paddy fields of Fujian province. Thus, the main objectives of the present study are (1) to determine the concentrations of heavy metals (Cd, Cr, Pb, As, Cu, Ni and Co) in paddy fields in Fujian province, (2) to evaluate the spatial distributions of heavy metals in paddy fields in Fujian province, and (3) to assess the soil heavy metal pollution levels and ecological risks in paddy fields in Fujian province.

## Material and methods

### Study area

**Figure 1:**
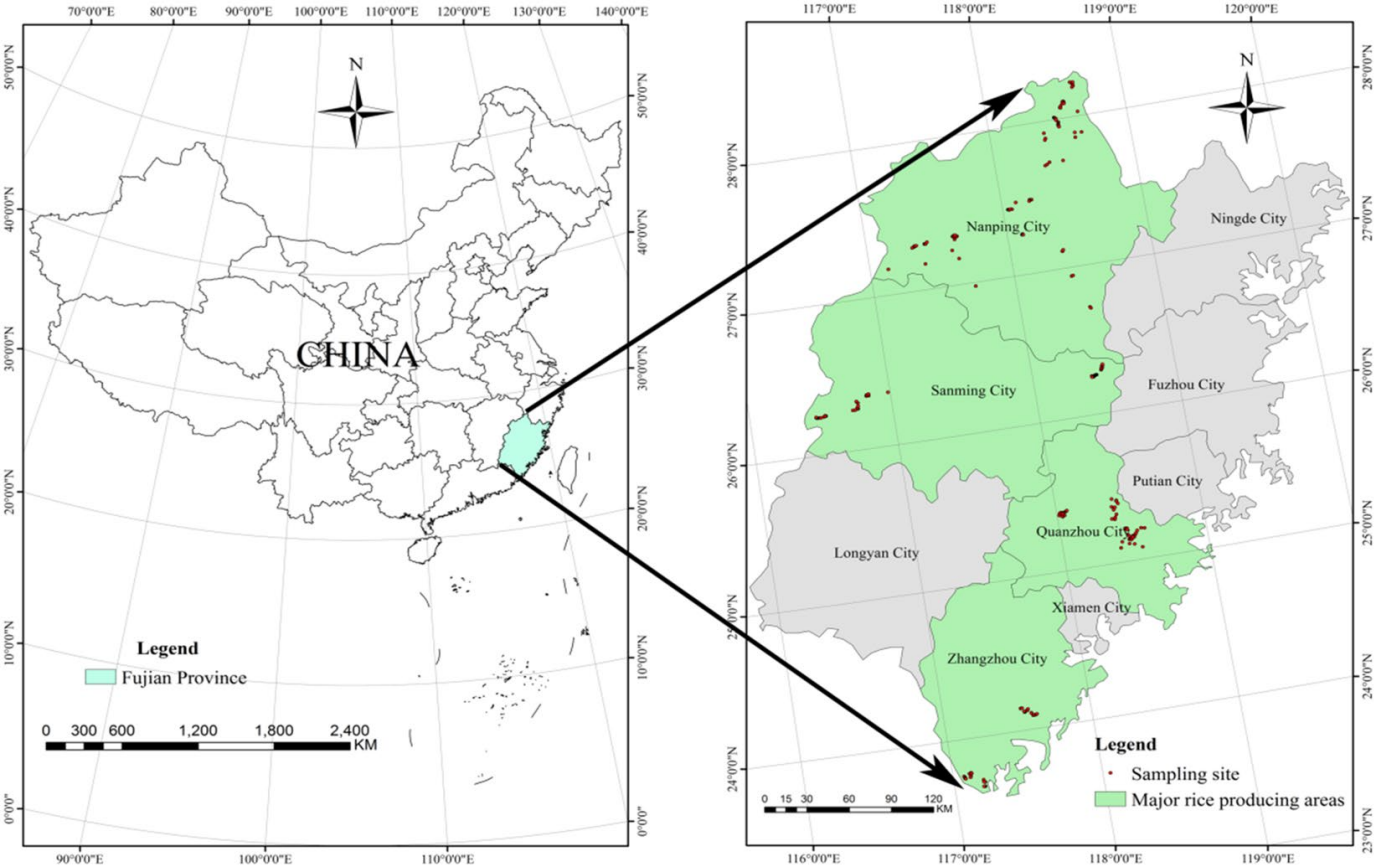
Location map showing the study area and sampling sites. (By the software of ArcGIS, version 10.2).

Fujian province is situated in southeast of China (23° 30′–28° 20′ N, 115° 40′–120° 30′ E), which is one of many mountainous provinces in China, with over 90% of the area covered with mountains and hilly lands (Fig. 1). It also belongs to the subtropical zone, with abundant sunshine, abundant heat and precipitation.

At the same time, the main region of rice cultivation in Fujian province is divided into northern and southern areas (Fig. 1), including Nanping city, Sanming city, Quanzhou city and Zhangzhou city, which make up more than 60% of the total rice planting area of Fujian province. In addition, according to the Fujian Statistical Yearbook (2017), Jianou city (county-level), Jianyang city (county-level), Pucheng county, Shaowu city (county-level), Ninghua county, Youxi County, Nanan city (county-level), Yongchun county, Zhangpu county and Zhaoan county are the main counties of rice cultivation in these cities.

### Data collection and preparation

#### Sample collection

A total of 272 soil samples were collected from the main counties of rice cultivation in Fujian province in July 2017 (Fig. 1). Each sample contained a pool of five subsamples that were taken from the cultivated horizon (0–20 cm) in a 20 × 20 m area, and the exact longitude and latitude of the sampling sites was recorded based on the global positioning system (GPS). The soil samples were air-dried after collection and then crushed to pass a 0.15 mm sieve prior to analysis.

#### Sample treatment and analysis

For various heavy metals measurements, the cadmium (Cd), chromium (Cr), lead (Pb), arsenic (As), copper (Cu), nickel (Ni) and cobalt (Co) were determined in this study. To measure the concentration of metals, 0.5 g soils from each sample were digested with a mixture of concentrated HCl–HNO_3_–HF–HClO_4_ on a hot plate^15,28^. First, a soil sample (0.5 g) was weighed and digested with 5 ml HCl on an electric hot plate at 190 °C until the solution was reduced to 3 ml. Second, approximately 5 ml HNO_3_, 5 ml HF, and 3 ml HClO_4_ were added to the solution and were digested until no black material remained. After cooling, the digestion solution was moved to a volumetric flask and was diluted to 50 ml. Finally, the digestion solution was filtered through a Millipore filter (0.45 μm), and then the concentration of heavy metals was analyzed by using inductively coupled mass spectrometry (ICP-MS, NexION 300X, Perkin Elmer, Melville, NY, USA). The quality control (reference material: GSB07-3272-2015 (ESS-5) and blank samples were generated. The recovery rates of Cd, Cr, Pb, As, Cu, Ni and Co in the reference material were 92%, 93%, 104%, 96%, 92%, 89% and 86%, respectively. The soil pH was also analyzed using a pH meter (Seven Compact, Mettler–Toledo, Greifensee, Switzerland) in 2.5:1 water/soil suspensions in this study.

### Spatial distribution of heavy metals

The location map and spatial distribution patterns of heavy metals were obtained by conducting geostatistical analyses with geographic information system (GIS) software (ArcGIS, version 10.2). In addition, the ordinary kriging method and the spherical model were used to analyze the spatial distribution of heavy metals.

### Assessment methods for heavy metals

#### Geo-accumulation method

The geo-accumulation index (${I}_{geo}$) has been widely used to assess the contamination levels of heavy metals in soil pollution by Müller in 1969, which enables the assessment of contamination by comparing current and pre-industrial concentrations^29,30^. In this study, the ${I}_{geo}$ was computed by using the following equation:

$${I}_{geo}={log}_{2}\frac{{C}_{n}}{1.5{B}_{n}}$$

where ${C}_{n}$ was the measured concentration of metal $n$ in paddy soil; ${B}_{n}$ was the geochemical background content of metal $n$ in the soil of Fujian province (Cd = 0.054 mg/kg, Cr = 41.30 mg/kg, Pb = 34.9 mg/kg, As = 5.78 mg/kg. Cu = 21.6 mg/kg, Ni = 13.5 mg/kg, Co = 7.41 mg/kg)^31^; the constant 1.5 was the factor compensating for the geochemical background content due to lithogenic actions. The geo-accumulation index consists of seven grades or classes, and data were classified as showed in the Table 1.

**Table 1:** The ${I}_{geo}$ classes in relation to soil quality.

${I}_{geo}$ classes	Soil quality
< 0	Practically uncontaminated
0–1	Uncontaminated to moderately contaminated
1–2	Moderately contaminated
2–3	Moderately to heavily contaminated
3–4	Heavily contaminated
4–5	Heavily to extremely contaminated
> 5	Extremely contaminated

#### Nemerow integrated pollution method

According to the previous studies^30–32^, the nemerow integrated pollution index (${P}_{n}$) was introduced as an integrated indicator to assess the quality of the soil environments. It could better reflect to the maximum and average concentration of heavy metals. In this study, ${P}_{n}$ was computed by using the following equation:

$${P}_{n}=\sqrt{\frac{{\left(\frac{{C}_{i}}{{S}_{i}}\right)}_{max}^{2}+{\left(\frac{1}{n}{\sum }_{i=1}^{n}\frac{{C}_{i}}{{S}_{i}}\right)}^{2}}{2}}$$

where ${C}_{i}$ was the measured concentration of metal $n$ in paddy soil, and ${S}_{i}$ was the standard value of metal $n$ in chinese environmental quality standards for acidic agricultural soils (GB 15618-1995; 0.30 mg/kg for Cd, 250.00 mg/kg for Cr, 250.00 mg/kg for Pb, 30.00 mg/kg for As, 50 mg/kg for Cu, 40 mg/kg for Ni). However, because there is currently no set standard for Co, the Fujian province soil background value was used to carry out the computation (7.41 mg/kg for Co) in this study. The nemerow integrated pollution index consists of five classes was showed in the Table 2.

**Table 2:** The ${P}_{n}$ classes in relation to soil quality.

${P}_{n}$ classes	Soil quality
< 0.7	No pollution
0.7–1	Warning line of pollution
1–2	Low level of pollution
2–3	Moderate level of pollution
> 3	High level of pollution

#### Potential ecological risk method

The potential ecological risk index ($RI$) was widely introduced to assess the degree of potential ecological risks of heavy metals in soil and in water, which was originally used in sediment by Hakanson^2,33^. In this study, the $RI$ was computed by using the following equation:

$${C}_{r}^{i}={C}^{i}/{C}_{n}^{i}$$

$${E}_{r}={T}_{r}^{i}{C}_{r}^{i}$$

$$RI={\sum }_{i=1}^{n}{T}_{r}^{i}{C}_{r}^{i}={\sum }_{i=1}^{n}{T}_{r}^{i}{C}^{i}/{C}_{n}^{i}$$

where $RI$ is the total potential ecological risk of individual heavy metal; ${E}_{r}$ is the potential ecological risk of individual heavy metal; ${T}_{r}^{i}$ is the heavy metal toxic-response factor (Cd = 30, Cr = 2, Pb = 5, As = 10, Cu = 5, Ni = 5, Co = 5)^34^; ${C}_{i}$ is the measured concentration of metal $n$ in paddy soil; and ${C}_{n}^{i}$ is the standard value of metal $n$ in chinese environmental quality standards for acidic agricultural soils (GB 15618-1995; 0.30 mg/kg for Cd, 250.00 mg/kg for Cr, 250.00 mg/kg for Pb, 30.00 mg/kg for As, 50 mg/kg for Cu, 40 mg/kg for Ni). However, because the national standard does not have a limit for the content of Co, the background value Fujian province soil was selected for computation (7.41 mg/kg for Co) in this study. The degree of ${E}_{r}$ was classified in five classes (Table 3). The degree of RI was classified as four classes (Table 4).

**Table 3:** The ${E}_{r}$ classes in relation to soil quality.

${E}_{r}$ classes	Soil quality
< 40	Low risk
40–80	Moderate risk
80–160	Considerable risk
160–320	High risk
> 320	Extreme risk

**Table 4:** The $RI$ classes in relation to soil quality.

$RI$ classes	Soil quality
< 150	Low risk
150–300	Moderate risk
300–600	Considerable risk
> 600	High risk

All the statistical analyses were conducted using statistical software package SPSS 20.0 (IBM SPSS Inc., Chicago, USA) for Windows. Before performing the correlation test, the data was checked for normality of distribution by Kolmogorov–Smirnov test. The correlation between the soil heavy metals and pH using Pearson’s correlation coefficients (*p* < 0.05) were considered indicate statistical significance.

## Results and discussion

### General characteristics of heavy metals in paddy field

The general characteristics of the heavy metals, Cd, Cr, Pb, As, Cu, Ni and Co in the paddy field of Fujian province are listed in Table 5. According to the chinese environmental quality standards for acidic agricultural soils (GB 15618-1995), the mean concentrations of all heavy metals (i.e., Cd, Cr, Pb, As, Cu, Ni and Co) had not exceeded their national standards, which were 0.26 mg/kg, 49.08 mg/kg, 91.94 mg/kg, 22.60 mg/kg, 27.98 mg/kg, 27.46 mg/kg and 10.57 mg/kg, respectively. However, the mean concentrations of these heavy metals were higher than their corresponding background values of Fujian province. Among them, the mean concentrations of Cd, Pb, As and Ni exceeded the background levels by 5.20, 2.63, 3.85 and 2.03 times, respectively.

**Table 5:** Statistical characteristics of soil heavy metal of paddy fields in Fujian Province.

HMs	Mean (mg/kg)	SD (mg/kg)	Max (mg/kg)	Min (mg/kg)	CV (%)	Point exceeded rate (%)	Background value (mg/kg)	Standard value (mg/kg)
Cd	0.26	0.12	1.02	0.06	45.02	32.72	0.05	0.30
Cr	49.08	37.12	283.38	6.58	75.62	0.37	41.3	250
Pb	91.94	41.03	312.31	34.36	44.63	0.74	34.9	250
As	22.60	7.99	69.44	9.22	35.35	15.07	5.87	30
Cu	27.98	14.99	113.91	6.18	53.56	9.19	21.6	50
Ni	27.46	15.33	77.72	7.56	55.83	16.54	13.5	40
Co	10.57	4.68	30.41	2.46	44.28	–	7.41	–

In paddy fields of Fujian province, all the max concentrations of Cd, Cr, Pb, As, Cu, Ni and Co exceeded the national standards (GB 15618-1995), which reached 1.02 mg/kg, 283.38 mg/kg, 312.31 mg/kg, 69.44 mg/kg, 113.91 mg/kg, 77.72 mg/kg and 30.41 mg/kg, respectively. In detail, the over-standard rates of Cd, Cr, Pb, As, Cu and Ni in the total soil samples were 32.72%, 0.37%, 0.74%, 15.07%, 9.19% and 16.54%, respectively. This may indicate that some of the soil samples in the paddy field of Fujian province were polluted.

The coefficient of variation (CV) can indicate the degree of variation in soil sampling sites, and the CV values for these metals (Cd, Cr, Pb, As, Cu, Ni and Co) increased in the following order: As (35.35%) < Co (44.28%) < Cd (45.02%) < Pb (44.63%) < Cu (53.56%) < Ni (55.83%) < Cr (75.62%).

Table 6 showed the correlation coefficients between heavy metals and the soil pH of paddy fields in this study. It can be seen that the correlation coefficients had very significant positive correlations between Co, Ni, Cu and Cd to Cr; Cd and Pb to Cu; Cu and Cd to Ni; Cu and Ni to Co; and Cd to Pb, which indicated that these dominant contaminants could be largely derived from the same sources and spreading processes. Similar mechanisms also be involved in the patterns seen in the previous study, which found that Cd–Cu had significant positive correlations^35^. However, it is interesting that As is not related to any other metals, which indicates that its pollution source is different from other metals.

**Table 6:** Correlation coefficient between heavy metals and the soil pH of paddy fields in Fujian Province.

	pH	Cr	Co	Ni	Cu	As	Cd	Pb
pH	1							
Cr	− 0.236**	1						
Co	− 0.159**	0.575**	1					
Ni	− 0.265**	0.827**	0.641**	1				
Cu	− 0.099	0.614**	0.605**	0.657**	1			
As	− 0.077	− 0.036	0.043	− 0.003	0.069	1		
Cd	− 0.053	0.171**	0.103	0.229**	0.219**	− 0.090	1	
Pb	− 0.031	0.057	0.100	0.104	0.263**	− 0.079	0.214**	1

**Significant at P < 0.01.

Meanwhile, the mean value of the soil pH of paddy fields was 5.51 in Fujian province, which indicated that the paddy field had acidic agricultural soils. For the correlation coefficient, the soil pH had a very significantly negative correlation with Cr, Co and Ni, which may suggest that the concentrations of Cr, Co and Ni will increase if the value of soil pH decreases.

### Spatial distribution of heavy metals in paddy fields

**Figure 2:**
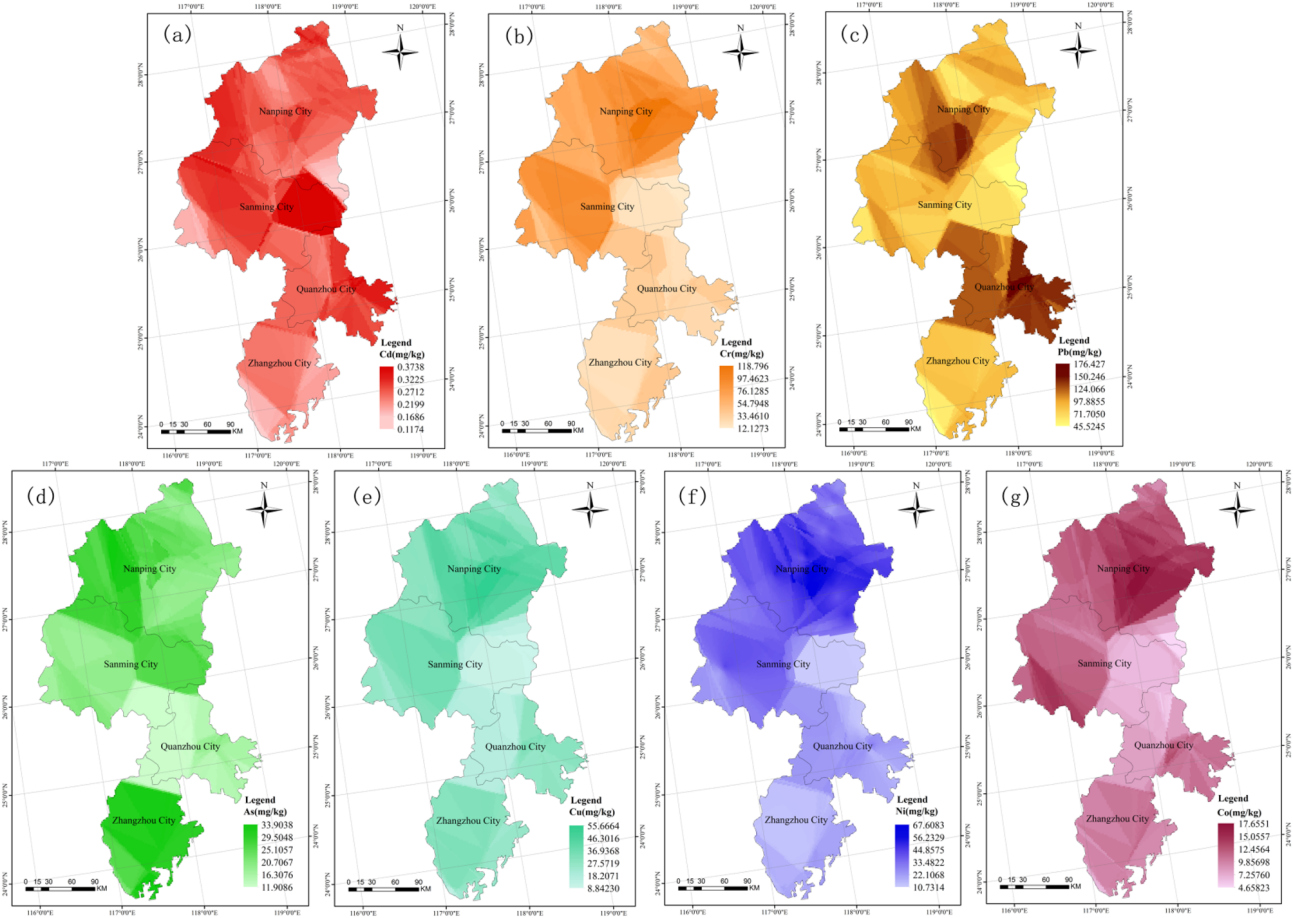
Spatial distribution map of heavy metals in paddy fields in Fujian province, China (By the software of ArcGIS, version 10.2).

The spatial distributions of heavy metals of paddy fields in Fujian province are shown in Fig. 2. The highest concentrations of Cd were found in southeast Sanming city and northeast Quanzhou city (Fig. 2a). For Cr, the areas with the highest content were located in southwest Sanming city and southeast Nanping city (Fig. 2b). The Pb hotspots were mainly in southeast Quanzhou city (Fig. 2c).

However, the highest contents of As, Cu and Ni were found in Zhangzhou city, the central region of Nanping city and southeast Nanping city, respectively (Fig. 2d–f). Finally, the highest content of Co was found in southeast Sanming city and southeast Nanping city (Fig. 2g). In general, there were substantial fluctuations in the spatial distribution of heavy metals in paddy fields in Fujian province.

### Contamination levels and the relevant ecological risks

**Figure 3:**
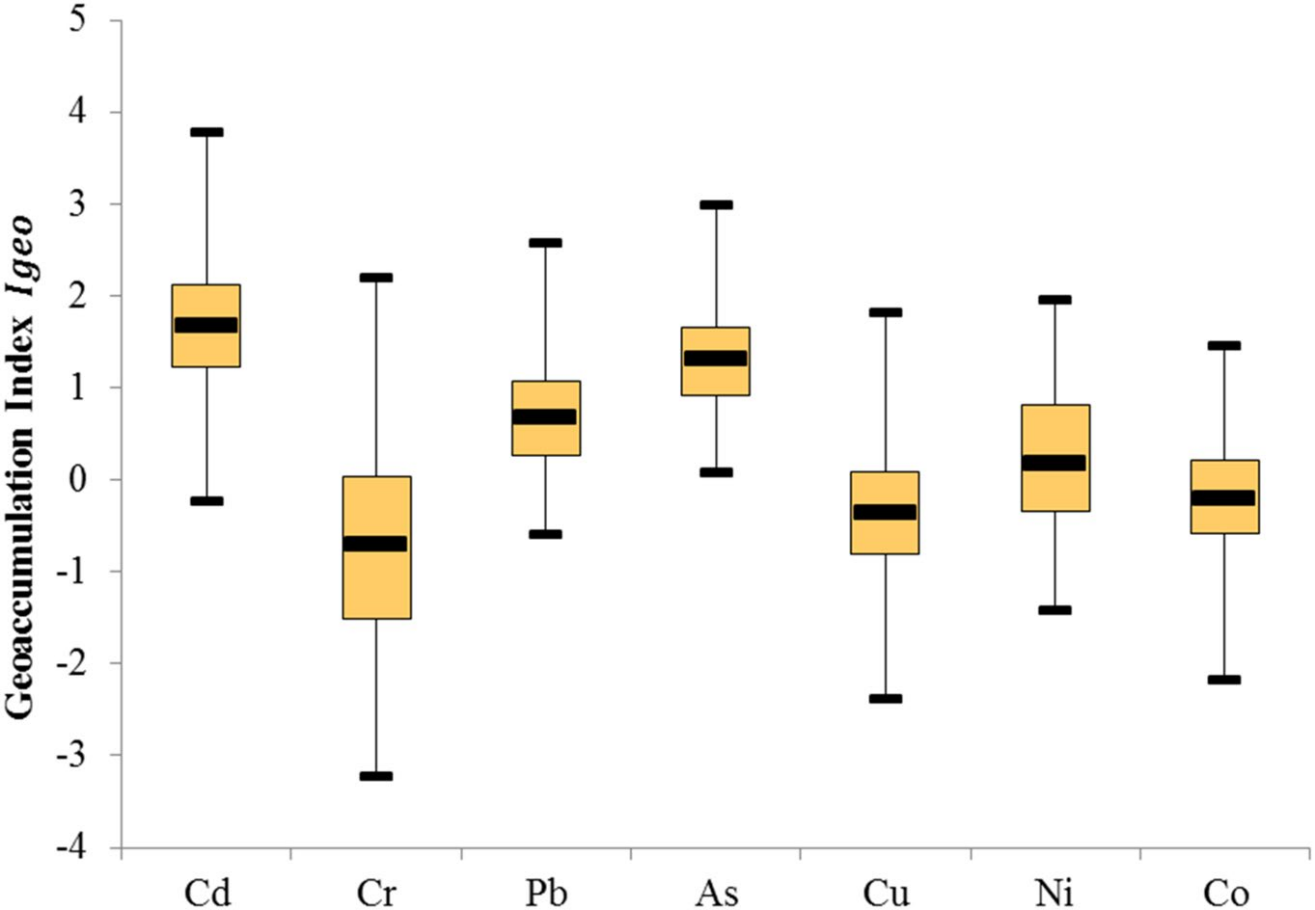
Box-plots of the Geo-accumulation index for the heavy metals of paddy fields in Fujian Province.

The calculated geo-accumulation index (${I}_{geo}$) values of heavy metals in paddy field are presented in Fig. 3. The mean values of ${I}_{geo}$ increased in the following order: Cr (− 0.70) < Cu (− 0.41) < Co (− 0.21) < Ni (0.24) < Pb (0.69) < As (1.27) < Cd (1.66). Meanwhile, the ${I}_{geo}$ values range from − 0.23 to 3.77 for Cd, − 3.24 to 2.19 for Cr, − 0.61 to 2.58 for Pb, 0.07–2.98 for As, − 2.39 to 1.81 for Cu, − 1.42 to 1.94 for Ni, and − 2.18 to 1.45 for Co.

In general, the mean values of Cd and As were indicative of moderately contaminated conditions, and the mean values of Pb and Ni were indicative of uncontaminated to moderately contaminated conditions. However, the mean values of Co, Cu and Cr showed that the paddy soils were practically uncontaminated in these three metals.

The nemerow integrated pollution index (${P}_{n}$) values of heavy metals in paddy fields are shown in Table 7. It is noted that the ${P}_{n}$ values of heavy metals were 1.12 for Fujian province, which belongs to a low level of pollution. Meanwhile, it is interesting that the ${P}_{n}$ values of heavy metals were 1.64 for Jianou city, 1.38 for Jianyang city, 1.20 for Pucheng county, 1.26 for Shaowu city, 1.29 for Ninghua county, 1.15 for Nanan city and 1.05 for Zhangpu county, which belong to a low level of pollution. However, the ${P}_{n}$ values for Yongchun county and Zhaoan county were 0.69 and 0.88, respectively, which indicated that these counties are located in the warning line of pollution. For Youxi county, the ${P}_{n}$ values were lowest (0.69) in all ten counties, which means that this county had a no pollution level.

**Table 7:** The nemerow integrated pollution index of heavy metal of paddy fields in Fujian province.

Area	${P}_{i}$							${P}_{N}$	Pollution level
	Cd	Cr	Pb	As	Cu	Ni	Co		
Fujian	0.86	0.20	0.37	0.75	0.56	0.69	1.43	1.12	Low level
Jianou	0.95	0.43	0.37	0.68	0.93	1.36	2.11	1.64	Low level
Jianyang	0.66	0.27	0.24	0.90	0.59	0.93	1.79	1.38	Low level
Pucheng	0.96	0.21	0.36	0.73	0.57	0.76	1.53	1.20	Low level
Shaowu	0.97	0.27	0.45	0.95	0.70	0.99	1.57	1.26	Low level
Ninghua	0.79	0.30	0.32	0.61	0.59	0.73	1.67	1.29	Low level
Youxi	0.85	0.06	0.23	0.84	0.21	0.28	0.84	0.69	No pollution
Nanan	1.04	0.13	0.55	0.56	0.57	0.55	1.46	1.15	Low level
Yongchun	0.93	0.11	0.50	0.45	0.44	0.47	1.05	0.84	Warning line
Zhangpu	0.76	0.12	0.34	1.02	0.57	0.46	1.33	1.05	Low level
Zhaoan	0.56	0.10	0.26	0.88	0.46	0.45	1.11	0.88	Warning line

**Figure 4:**
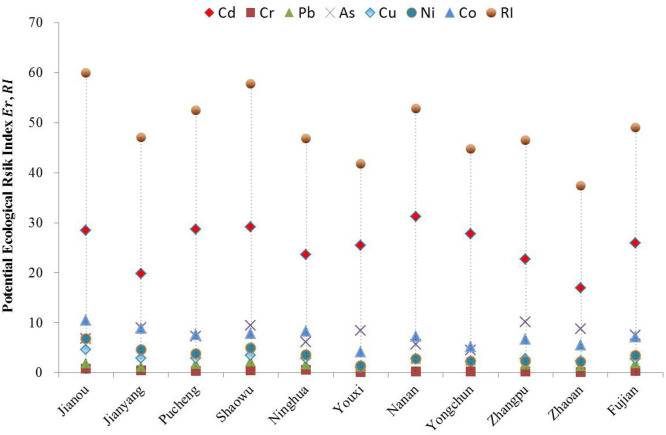
The potential ecological risks of heavy metals of paddy fields in Fujian Province.

The potential ecological risk index ($RI$) of seven heavy metals (i.e., Cd, Cr, Pb, As, Cu, Ni and Co) in paddy fields and the potential ecological risk index ($RI$) of total heavy metals are shown in Fig. 4. According to grading standards, the single potential ecological risk index (${E}_{r}$) values for no pollution soil (Low risk) were lower than 40, and the $RI$ was lower than 150. In Fujian province, the $RI$ value was 49.06, which suggested a low risk. For Cd, Cr, Pb, As, Cu, Ni and Co, the ${E}_{r}$ values were 25.94, 0.39, 1.84, 7.53, 2.80, 3.43 and 7.13, respectively, which suggested a low risk in paddy fields.

For counties, the results indicated that, in paddy fields, the Cd concentration contributed to the majority of potential ecological risks in these ten counties in Fujian province, which are presented in descending order: Jianou city (28.44) > Shaowu city (29.20) > Pucheng county (28.73) > Jianou city (28.44) > Yongchun county (27.76) > Youxi county (25.49) > Ninghua county (23.63) > Zhangpu county (22.68) > Jianyang city (19.83).

## Discussion

In this research, we found that Fujian province’s mean concentrations of heavy metals (Cd, Cr, Pb, As, Cu, Ni and Co) in paddy fields were greater than the background level in soils, and the max values of these metals were higher than the National Environmental Quality Standards for acidic agricultural soils (GB 15618-1995). However, the mean values of these metals in paddy fields were lower than the National Environmental Quality Standards. Some soil samples of paddy fields indicated that Fujian province had been polluted by Cd, Cr, Pb, As, Cu, Ni and Co. This result showed a similar pattern to that of Du et al., which found that 57.5% of soil samples in paddy field of Hunan province exceed the allowable limit specified by the china soil environmental quality standards^18^. According to the studies that are mentioned above^19,20,23,28^, the agricultural soil is mainly contaminated by Cd, Cu, Pb and Zn to various degrees in southern China. Meanwhile, compared with the pollution survey in the Yangtze river delta and the Pearl river delta^1,17–20,26,35^, we found that Fujian province contains a low level of heavy metal pollution, which is mainly due to the low degree of industrialization and the long-term development of the green economy in the region, such as construction of an ecological province in 2000.

However, it is interesting that 32.72% of the total soil samples were polluted by Cd in Fujian province, and they were located mostly in Nanan city, where there are many industrial factories, mining activities and high-density transports. Meanwhile, some soil samples in Zhangzhou city were contaminated by As, where high-intensity agricultural cultivation and many pesticides (1.03 × 10^4^ t) and fertilizers were used, such as 1.03 × 10^4^ t and 37.55 × 10^4^ t of pesticides and fertilizers were consumed in 2017. Therefore, it is important to identify the potential sources of heavy metals in future research for the balance management of heavy metals in paddy fields and safe production processes of rice in Fujian province.

The geo-accumulation index (${I}_{geo}$), nemerow integrated pollution index (${P}_{n}$) and potential ecological risk ($RI$) were indicated the low risk of paddy field in Fujian province. However, the Cd should be noted because it poses most of the potential ecological risks in Fujian Province. Therefore, if certain areas are contaminated by heavy metals, phytoremediation can be performed to remove heavy metals from contaminated soil and promote the health and sustainable development of agricultural ecosystems^36,37^.

## Conclusion

Our study showed that the overall quality of paddy fields in Fujian provinces is relatively safety, although some points exceed the National Environmental Quality Standards of China. Due to the terms of space, concentrations of Cd, Cr and Co from Sanming City in the rice fields, and Cu and Ni from Nanping in rice fields were similar, respectively. But, the concentrations of Pb and As in rice fields from Quanzhou and Zhangzhou were different. The multiple geographic analysis indices (${I}_{geo}$, ${P}_{n}$ and $RI$) was showed the low risk of paddy field in Fujian province. However, the Cd should be noted because it poses most of the potential ecological risks (${E}_{r}$ = 25.09) in Fujian province.
